# Oral Microbiota in Severe Early Childhood Caries in Thai Children and Their Families: A Pilot Study

**DOI:** 10.3389/fmicb.2018.02420

**Published:** 2018-10-15

**Authors:** Ruth G. Ledder, Kanokporn Kampoo, Rawee Teanpaisan, Andrew J. McBain

**Affiliations:** ^1^Division of Pharmacy and Optometry, School of Health Sciences, Faculty of Biology, Medicine and Health, The University of Manchester, Manchester, United Kingdom; ^2^Faculty of Dentistry, Prince of Songkla University, Songkhla, Thailand

**Keywords:** severe early childhood caries, Thailand, DNA profiling, familial homology, *Streptococcus mutans*, *Lactobacillus*, multi dimensional scaling

## Abstract

Thailand has a comparatively high prevalence of severe early childhood caries (S-ECC). S-ECC adversely affects the quality of life for children and their caregivers and represents a considerable economic burden. We have assessed the bacteriological composition of unstimulated saliva, dental plaque, and degraded dentine in a Thai cohort, including children with S-ECC and children without cavities; their siblings, and their primary caregivers. Samples were collected during a dental examination and patients were scored for plaque accumulation and their decayed, missing, and filled teeth (dmft) index. Samples were analyzed using differential bacteriological counting and gel-based eubacterial DNA profiling. Plaque *Lactobacillus* abundance correlated significantly with S-ECC. Whilst *Lactobacillus* counts were significantly higher in children with S-ECC than in their siblings and primary caregivers (five families), the opposite trend was apparent for cavity-free children. Counts of Gram-negative anaerobes were significantly lower in children with S-ECC than orally healthy children. S-ECC correlated significantly with plaque index scores, dmft, and with *Lactobacillus* abundance in a highly predictive manner. DNA profiles showed significant homology between families but not within non-cavity and S-ECC groups. In conclusion, salivary and plaque *Lactobacillus* counts were significantly associated with S-ECC in the Thai subjects. *Lactobacillus* counts in the children were not correlated with those of their siblings and primary caregivers. Individuals could be significantly differentiated based on family but not on caries status.

## Introduction

Early childhood caries is a common chronic condition affecting the primary dentition, which may progress to severe early childhood caries (S-ECC), resulting in the partial or complete destruction of the primary dentition (Davies, 1998; Drury et al., 1999; Ismail and Sohn, 1999; Vadiakas, 2008). Several previous investigations have demonstrated a comparatively high prevalence of S-ECC in Thailand, with one study reporting incidence rates as high as 44.5% for non-cavitated lesions (initial lesions) and 24.5% for cavitated lesions (advanced lesions) in 15–19 month-old children in central Thailand (Vachirarojpisan et al., 2004). A longitudinal study of 599 children with S-ECC in Southern Thailand indicated that caries incidence was 2.0% at 9 months of age, 22.8% at 12 monthsand 68.1% at 18 months (Thitasomakul et al., 2006) illustrating the rapid progress of this condition.

Early childhood caries is defined as the presence of one or more decayed, missing, or filled tooth surfaces in any primary tooth (dmft greater than zero) of children younger than 6 years old whereas S-ECC is defined as the presence of any smooth surface dental caries in children younger than 3 years old (Vadiakas, 2008). Previous studies have attempted to understand the microbiological etiology of early childhood caries using various analytical approaches (Salako et al., 2004; Corby et al., 2005; Rozkiewicz et al., 2006; Aas et al., 2008; Gizani et al., 2009). For example, Aas et al. (2008) amplified, cloned, and sequenced 16S rRNA genes and, together with reverse-capture checkerboard DNA–DNA hybridization, analyzed patients recruited from Columbus, OH, United States from a diverse age range (2–21 years old). *Capnocytophaga granulosa*, *Eubacterium*, *Streptococcus cristatus*, and *S. sanguinis* were detected at the same levels in plaque adjacent to intact enamel of the primary teeth of subjects with caries and in caries-free subjects (Becker et al., 2002; Aas et al., 2008). These observations are consistent with another previous report where *Actinomyces gerencseriae*, bifidobacteria, *Streptococcus mutans*, veillonella, *S. salivarius*, *S. constellatus*, *S. parasanguinis*, and *Lactobacillus fermentum* were associated with caries (Becker et al., 2002).

Most studies of childhood-associated dental caries have examined the bacterial composition in primary and permanent teeth (Minah and Loesche, 1977; Becker et al., 2002; Rozkiewicz et al., 2006; Aas et al., 2008). In a study that used PCR-DGGE, the overall microbial diversity in plaque samples was significantly greater in the control group than in the S-ECC group (Li et al., 2007a). Few studies have investigated any potential familial influences that could be mediated through the acquisition and exchange of oral bacteria between close relatives. Transmission of the causative organisms of S-ECC amongst families is likely to be an essential step in disease progression. A better understanding of this process could assist in the implementation of enhanced disease control and prevention strategies. We have therefore determined: (i) the composition and consortial similarity of saliva and supra-gingival plaque eubacterial communities in children with and without S-ECC, (ii) the comparison of bacterial composition and potential transmission within mother-child pairs and between siblings, and (iii) the composition and concordance of bacterial communities amongst different sites of S-ECC from the same patient.

## Materials and Methods

### Selection of Subjects

Approval for this study was provided by the Ethics Committee of the Faculty of Dentistry, Prince of at the Faculty of Dentistry, Prince of Songkla University. In all cases, informed consent was obtained from parents or primary caregivers before the study commenced. Volunteers comprised of cavity-free (CF) subjects who had no cavities (*F* = 1, *M* = 4) age range 31–85 months (mean 49.6) and those with S-ECC if age (<6 years) or caries (if age >6 years) (*F* = 2, *M* = 3) age range 31–84 months (mean 45.6) and their relatives. All volunteers were in good general health and clinical access to at least one sibling and primary caregiver was possible. Patients with documented systemic disease, physical or mental health problems, those who had received antibiotics or other drug therapy in the previous 3 months, and those with a history of hospitalization were excluded from the study. S-ECC was defined according to the American Academy of Pediatric Dentistry as outlined by Ismail and Sohn (Ismail and Sohn, 1999; AAPD, 2005). The plaque index was measured according to the methods of Ainamo and Bay (1975). All subjects were recruited consecutively and dental health examinations performed. Family members (at least one primary caregiver and one sibling) were also invited to participate in a dental examination. The incidence of caries was recorded according to the DMFT index in which D refers to the number of untreated carious teeth, F represents the filled teeth, and M refers to the number of extracted teeth due to caries. Teeth which were extracted due to periodontal diseases or orthodontic reasons were excluded from this index (WHO, 2000).

### Sampling

Unstimulated saliva from all patients and their family members was collected by expectoration into sterile Universal bottles, maintained on ice and carried from clinic to the laboratory in under 30 min for analyses. In all cases, a portion (c. 1 ml) of each saliva sample was archived at -60°C for subsequent analysis. Dental plaque samples were freshly obtained in all cases. Supragingival plaque from healthy subjects was sampled from a minimum of four sites, including at least two anterior and posterior teeth, and the samples were pooled. Pooled plaque from caries subjects was collected separately from two types of sites: (i) from the surfaces of healthy enamel and (ii) from the surfaces of cavitated lesions Plaque samples were collected separately using a sterile dental spoon excavator EXC 23 (Sci-Dent, Inc., Hamburg, NY, United States), sterile dental spoon excavator No. 40–41 (American Eagle Instruments^®^ INC., Missoula, MT, United States) and a sterile discoid cleoid CD 89/92 (Hu-Friedy Mfg. Co., Inc., Rockwell City, IA, United States). These three instruments had the same blade size. Once collected, plaque samples were put into sterile Eppendorf tubes and weighed before use. For bacteriologic analyses, plaque was then transferred to half-strength thioglycolate broth (1 m) for serial dilution. Samples were archived at -60°C for subsequent analysis. Excavated carious dentine from cavitated lesions was also collected after rinsing debris. The sample sites were isolated and dried with a rubber dam sheet and an air spray; and carious dentine was removed using sterile dental spoon excavators as outlined above. As for dental plaque samples, carious dentine samples were processed immediately and also archived at -60°C for subsequent analyses.

### Differential Bacteriological Analysis

For bacteriological enumeration, samples of saliva, dental plaque and dental caries were homogenized by vortexing for approximately 60 s. Sample homogenates were then serially diluted in sterile pre-reduced, half strength thioglycolate medium (USP). Appropriate dilutions (0.1 ml) were then plated in triplicate onto a variety of proprietary agar media to differentially isolate and enumerate various functional groups of oral bacteria. Wilkins Chalgren agar (WC) for total aerobes and total anaerobes, respectively; Wilkins Chalgren agar with Gram-negative supplement (WCGN); trypticase yeast extract cysteine sucrose agar (TYCS) (Van Palenstein Helderman et al., 1983; Schaeken et al., 1986) for streptococci and Rogosa agar (RA) for total lactobacilli. After plating, media were transferred immediately to an anaerobic cabinet (Don Whitley Scientific, Shipley, United Kingdom; gas mix 10:10:80; H_2_, CO_2_; N_2_) and incubated at 37 ± 0.5°C, with the exception of the total aerobe counts which were incubated in a bench-top incubator (Cole-Parmer, London, United Kingdom) at 37°C for up to 5 days.

### Bacteriological Analysis by PCR-DGGE

DNA was extracted from the archived samples using a DNA stool mini kit (Qiagen Ltd., West Sussex, United Kingdom) in accordance to manufacturer’s instructions and were analyzed by PCR-DGGE as previously described (McBain et al., 2003).

### Analysis of DNA Profiles

This was done as described by Kampoo et al. (2014). Briefly, negative images of stained DGGE gels were aligned using Adobe Photoshop CS6 (Adobe, London, United Kingdom) and then analyzed with Bionumerics v.5.1 (Applied Maths, Sint-Martens-Latem, Belgium). Lanes on gel images were selected manually and then compared to reference lanes. To test for potential differences in plaque or salivary composition, binary band matching profiles for each lane were analyzed with PRIMER software (v. 6) (Primer-E Ltd., Luton, United Kingdom) as follows. Bray-Curtis similarity values were calculated for imported binary gel band data and agglomerative hierarchical clustering was done via the CLUSTER menu of the PRIMER software. Similarity profile permutation tests were used to test for statistically significant evidence of genuine clusters and data were further analyzed by using the non-metric multi-dimensional scaling (MDS) algorithm. To test the significance of potential differences in bacteriological profiles, analysis of similarity (ANOSIM) was done with the ANOSIM test.

### Statistical Analyses

Means, standard deviations (SD) ranges (min-max) and/or frequencies were calculated using Microsoft Excel. The non-parametric, Mann–Whitney *U* test was used to identify significant difference of general data. The ANOVA with Scheffe test and paired *t*-test was used to compare mean bacterial counts from different sites of samples and amongst family members. Data were analyzed using Statistical Packaging for Social Sciences (SPSS). A forward correlation analyses constructed using SPSS version 16.0 to examine two relationships.

## Results

### Clinical and Behavioral Variables Associated With S-ECC

Clinical data for all volunteers and their families are shown in **Table 1**. Age was not a significant factor between the CF and S-ECC groups. Although the frequency of additional sugar consumption was not significantly different between CF and S-ECC children, a higher number of S-ECC subjects were reportedly given a bottle of milk overnight (*p* = 0.05) than CF subjects.

**Table 1:** Clinical and dietary data for cavity-free and severe early childhood caries patients and their families.

Variable	S-ECC (*n* = 5)	Siblings (*n* = 5)	CF (*n* = 5)	Siblings (*n* = 6)
Age in months (min-max)	41.2 (31–62)	108 (51–204)	49.6 (31–85)	70.3 (60–79)
Sex	F, 2; M, 3	F, 2; M, 3	F, 1; M, 4	F, 2; M, 4
Bottle overnight^∗^	60%	na	0%	na
Additional sweetened drinks	80%	na	80%	na
Plaque index^∗^	2.04 ± 0.61	0.83 ± 0.72	0.33 ± 0.37	0.50 ± 0.47
dmft^∗^	14.8 ± 1.94	5.0 ± 5.06	1.40 ± 1.50	1.50 ± 2.93


*CF, cavity-free; S-ECC, severe early childhood caries. ^∗^Significant (*p* < 0.05) difference between CF and S-ECC subjects dmft-decay, missing, filled teeth.*

Cavity-free subjects had plaque index scores 70% lower than individuals with S-ECC (determined on the basis of plaque accumulation). In addition, dmft and plaque indices were significantly higher (*p* < 0.05) in S-ECC than in CF children. There was no significant difference in caries experience scores (dmft) and plaque indices between the siblings of CF children and S-ECC children.

### Oral Bacteriological Profiles in Cavity-Free and S-ECC Children

**FIGURE 1:**
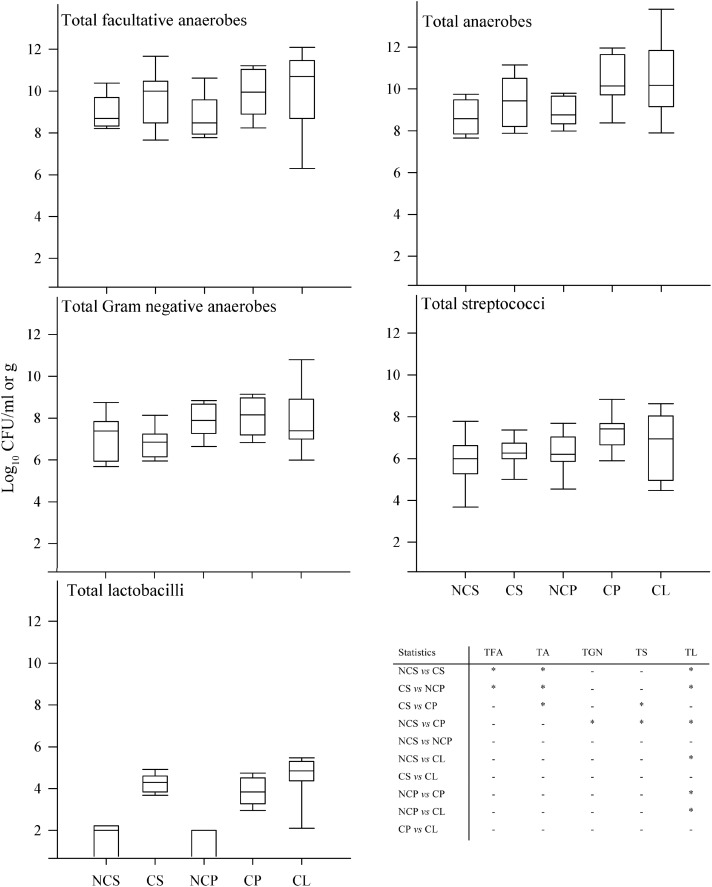
Differential bacterial composition from various sites in CF children and S-ECC patients. Total facultative anaerobes, total anaerobes, total Gram-negative anaerobes, total streptococci, and total lactobacilli; NCS (saliva from cavity free children), CS (S-ECC saliva), NCP (dental plaque from CF children), CP (S-ECC dental plaque), and cavitated lesion (Degraded dentine). Data were analyzed using ANOVA with the Scheffe test and significance was compared between sites within bacterial groups (^∗^*p* < 0.05).

Five major functional groups of oral bacteria: total facultative anaerobes, total anaerobes, total Gram-negative anaerobes, total streptococci, and total lactobacilli from saliva, supragingival plaque and degraded dentine were analyzed in samples from CF and S-ECC children; and from saliva obtained from their caregivers/siblings (**Figure 1**). Mean total counts for all samples of facultative anaerobes and anaerobes ranged between 8 and 10 Log_10_ CFU/ml, respectively. Gram-negative anaerobe numbers ranged between 6 and 10 Log_10_ CFU/ml while number of streptococci ranged between 4 and 9 Log_10_ CFU/ml. Lactobacilli were present in lower numbers (1–5 Log_10_ CFU/ml). There were significant differences (*p* < 0.05) in bacterial composition in saliva and in dental plaque between cavity-free children (CF) and children with S-ECC. Total facultative anaerobes and total anaerobes counts were significantly higher in children with S-ECC compared to CF individuals. For comparisons between counts in saliva and dental plaque, the numbers of total anaerobes, Gram-negative anaerobes and streptococci were markedly higher in S-ECC plaques than in the corresponding saliva samples. This trend was however, not manifested in CF children. Counts of total and lactobacilli were significantly higher (*p* < 0.05) in S-ECC plaques than in plaques from CF subjects whilst total lactobacilli were significantly higher in S-ECC saliva than in saliva from CF volunteers, and also higher in degraded dentine than in dental plaque from S-ECC children.

### DNA Profiles Derived From Saliva and Supra-Gingival Plaques Collected From Cavity-Free, S-ECC Patients, and Their Families

**FIGURE 2:**
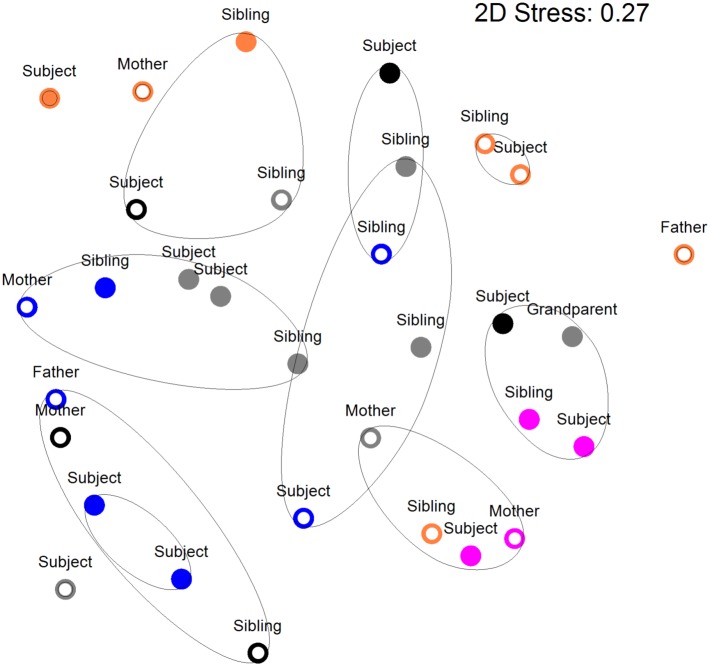
Salivary DNA profiles from primary subject cavity-free and S-ECC children (age <6 years) or caries (age >6 years), and caregivers and their siblings. Open circles, cavity–free; closed circles, S-ECC. Symbol color: Family 1, black; Family 2, purple; Family 3, blue; Family 4, orange; Family 5, gray. Contour lines on the MDS plot superimpose 50% resemblance levels derived from cluster analysis (not shown). Profiles could be differentiated on the basis of family (*p* < 0.05). S-ECC and cavity-free families could not be differentiated (*p* = 3.18). Carious sites and cavity-free sites could not be differentiated (*p* = 8.1).

**FIGURE 3:**
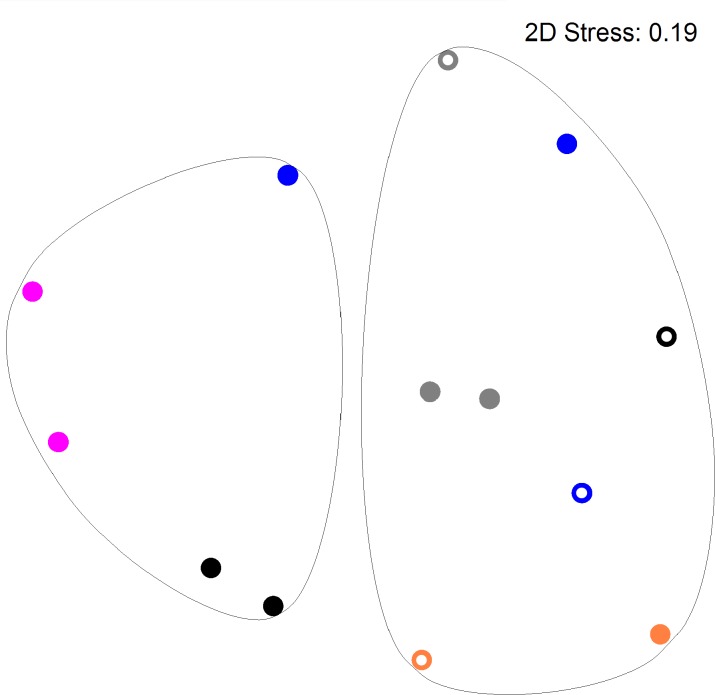
DNA profiles derived from supragingival plaque from S-ECC and cavity-free children (see legend to **Figure 2**). Profiles could be differentiated on the basis of family (*p* = 0.036). ECC and cavity-free families could not be differentiated (*p* = 4.18). Carious sites and cavity-free sites could not be differentiated (*p* = 7.38).

**FIGURE 4:**
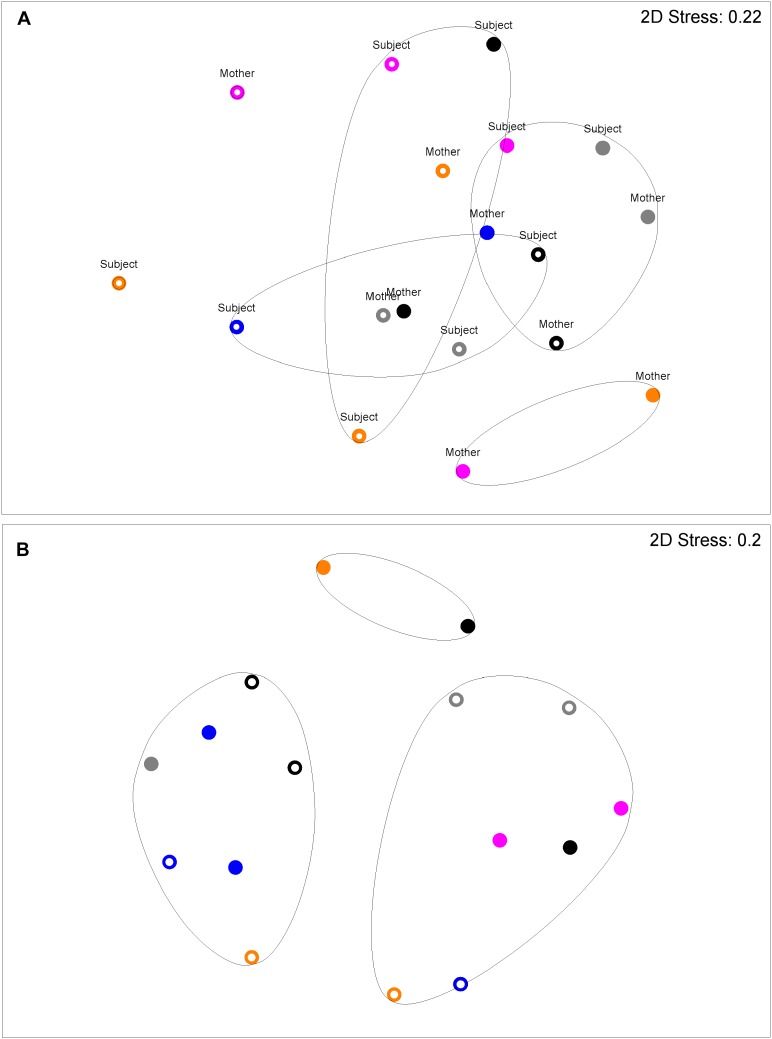
DNA profiles derived from saliva **(A)** and plaque **(B)** from S-ECC children and their caregivers (see legend to **Figure 2**). Salivary profiles associated with S-ECC and cavity-free families could be differentiated (*p* = 0.037). Profiles could not be differentiated on the basis of family (*p* = 4.72). Plaque profiles could not be differentiated on the basis of family (*p* = 0.10). S-ECC and cavity-free families could not be differentiated (*p* = 4.18). Carious sites and cavity-free sites could not be differentiated (*p* = 7.38).

**FIGURE 5:**
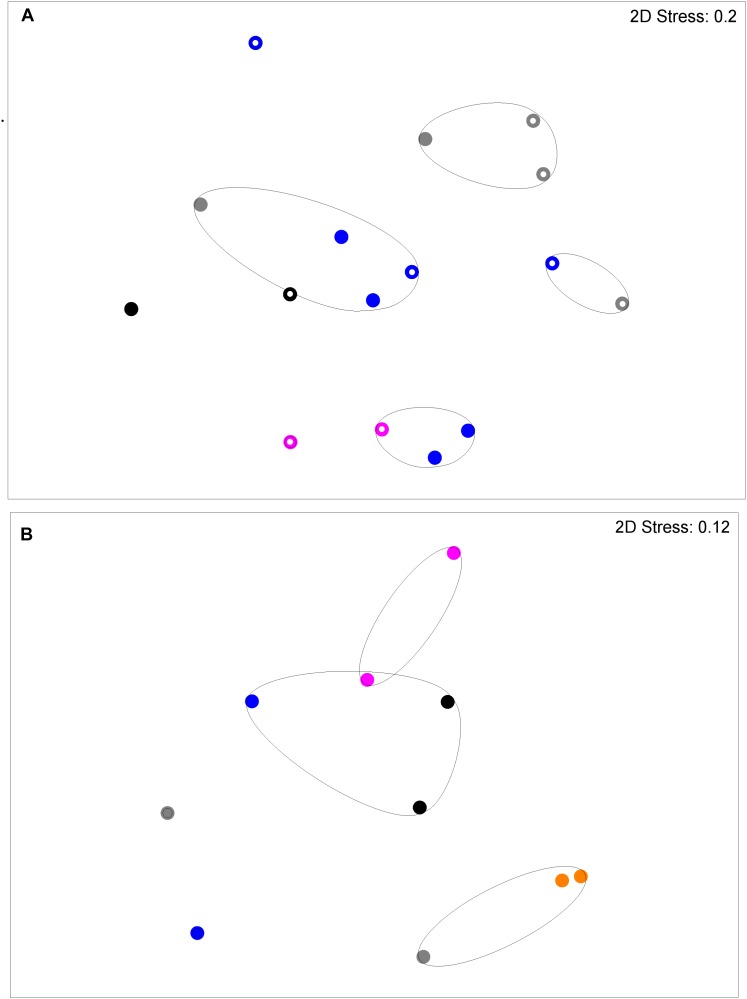
DNA profiles derived from plaque **(A)** and saliva **(B)** from S-ECC children and their siblings (see legend to **Figure 2**). Plaque profiles could be differentiated on the basis of family with (*p* = 0.031). S-ECC and cavity-free families could not be differentiated (*p* = 4.18). Carious sites and cavity-free sites could not be differentiated (*p* = 8.39). Salivary profiles could be differentiated on the basis of family (*p* = 0.02).

**Figure 2** shows a multi-dimensional plot (MDS) for saliva from primary subject CF, S-ECC children and their families. This analysis indicated that the eubacterial profiles of these patients’ samples clustered significantly (*p* < 0.05) on the basis of familial relatedness. There were no significant differences observed between S-ECC and CF families and between carious and CF sites. The MDS in **Figure 3** indicates that DNA profiles derived from the supragingival plaque of ECC and CF children could be significantly differentiated in terms of familial relatedness (*p* = 0.036) but could not, however, be differentiated in terms of caries status. The MDS profile generated from saliva (**Figure 4A**) of all children and their primary caregivers showed that S-ECC families were differentiated from CF families (*p* = 0.037) but not based on familial relatedness (*p* = 4.72). The supragingival plaque profiles (**Figure 4B**) of these families were now significantly differentiated based on relatedness and caries status. The siblings of S-ECC children were differentiated based on both plaque (**Figure 5A**) and saliva (**Figure 5B**).

### Associations of Bacterial Profiles Within Mother–Child Pairs and Between Siblings

**FIGURE 6:**
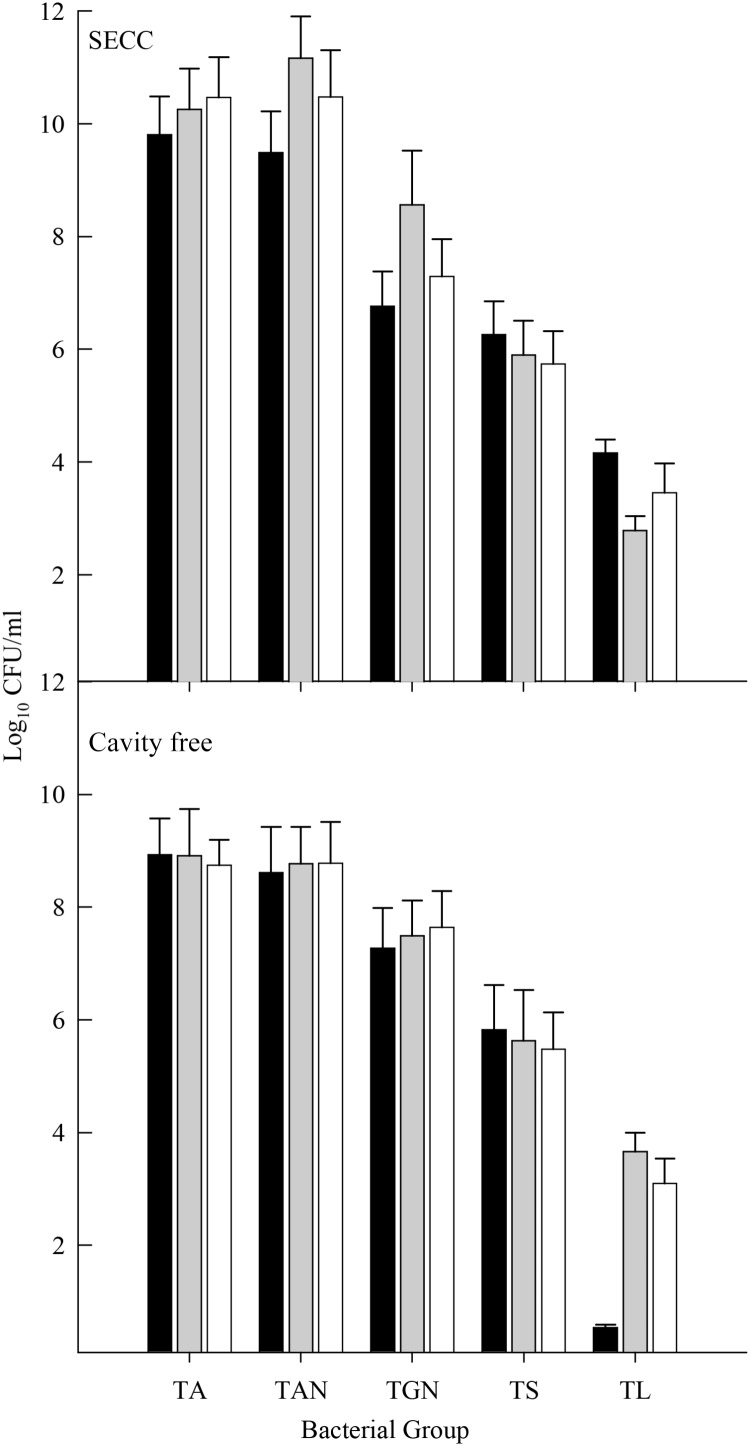
Salivary counts of total facultative anaerobes (TA), total anaerobes (TAN), total Gram-negative anaerobes (TGN), total streptococci (TS), and total lactobacilli (TL) in S-ECC and caries free individuals. Black bar, child subjects; gray bar, siblings and white bar, primary care giver. Data were analyzed using the one-way ANOVA with Scheffe test for significance within and between bacterial groups and within and between families. Significant differences (*p* < 0.05) in lactobacilli counts were found between cavity free children and their families and between S-ECC children and their family members for Gram-negative anaerobe counts.

**Figure 6** shows five major bacterial groups obtained from the saliva of CF and S-ECC family members. Counts of facultative anaerobes, anaerobes, and streptococci among family members of both CF and S-ECC children were not significantly different. Importantly, however, total lactobacilli were significantly lower (*p* < 0.05) in the CF children compared to their family members who had lactobacillus counts comparable to the family members of S-ECC children. Total Gram-negative anaerobe numbers were significantly lower in S-ECC children than in their family members (*p* < 0.05) (**Figure 6**). Therefore, in contrast to CF children, the salivary bacterial composition of S-ECC patients was characterized by comparatively low levels of Gram-negative anaerobes and markedly elevated lactobacilli.

### Statistical Analyses

Total lactobacilli numbers recovered from saliva and dental plaque were significantly (*p* < 0.05) different between CF and S-ECC groups. There were significant positive correlations (*p* < 0.05) in salivary facultative anaerobes and streptococci between primary caregivers and children. While there was no significant correlation in all groups of bacteria obtained from the dental plaque of both groups, there was a moderate positive correlation with respect to total facultative anaerobes, total streptococci and total lactobacilli (47–55%). A significant difference in bacterial counts between subjects and their siblings was only observed for salivary anaerobes (*p* < 0.05). However, the association between children and their siblings mostly showed positive correlations ranging from 14 to 88%. Significant differences (*p* < 0.05) between dmft and plaque index scores between CF children and S-ECC patients was observed. The association between dmft scores and bacterial counts were analyzed. There was a highly significant correlation (*p* < 0.05) between salivary lactobacilli, dental plaque lactobacilli, and plaque index scores; and between salivary lactobacilli, dental plaque lactobacilli and dmft scores. Moreover, highly significant correlations (*p* < 0.05) between dmft and plaque index scores and between d scores (decay score) and plaque index scores were also observed (>90%).

## Discussion

This cross-sectional pilot study aimed to better understand the etiology of S-ECC, in children living in Southern Thailand. The number of participants (*n* = 32) is in agreement with previous recommendations (Browne, 1995) for recruiting a representative sample size for a pilot trial. Indicative power calculations with data generated in the present study suggest that sample sizes between cavity and CF children required for 80% statistical power would be eight subjects for salivary total anaerobes, six subjects for streptococci in plaque and, due to the large difference in counts, only three subjects for salivary lactobacilli. A number of variables are known to be involved in the causation of dental caries (Marsh, 2003). These mostly relate to local acidification of dental enamel that is largely due to the metabolic activities of dental plaque (Marsh and Bradshaw, 1995). Several factors influence the rate and extent of acidification and thus the cariogenic process, which can be broadly divided into environmental and microbial factors. Simplistically, the degree of acidification depends on the types and quantities of fermentable carbohydrates that the individual consumes, but plaques may vary markedly in the rate by which they can produce acid and also in terms of the terminal pH reached at the tooth surface. Additionally the amount of plaque that accumulates varies considerably according to (i) the location of colonization; (ii) individuals dental hygienic practices; and (iii) diet.

In the current study, the contribution of a range of microbial and environmental factors of potential etiological importance were investigated in children suffering from S-ECC, but also the caregiver(s) and their sibling(s). These are outlined in **Table 1** and include plaque indices, which are a semi-quantitative clinical measure of plaque accumulation on the surfaces of teeth, and DMFT, a measure of current and past experience of dental caries. These were measured in the child (dmft) and also in their family members (DMFT) to give an indication of overall familial dental health. Environmental factors including the practice of giving the child a bottle of milk during the night and the use of supplemental sugar in drinks or other food were recorded. Since a number of molecular biological tools and analytical software packages have been recently developed for characterizing microbial communities to assist with comparing microbial profiles, these were used to assess the possible role of the transmission of bacteria from mother to child or between siblings. With respect to the S-ECC and CF control volunteers that were recruited; age and gender were similar; however, the history of overnight bottle use, plaque index and dmft scores were significantly (*p* < 0.05) greater in S-ECC subjects than in CF subjects. A previous study has also reported positive associations between children’s caries experience and a modified debris index score which is analogous to the plaque index (Vachirarojpisan et al., 2004), an observation further supported by a more recent clinical study (Jigjid et al., 2009). This supports the suggestion that effective mechanical plaque removal may reduce the incidence of dental caries in children.

There was a statistically significant association between numbers of total streptococci and S-ECC in dental plaque (**Figure 1**), which is in agreement with a previous study that reported an association between *S. mutans* and S-ECC (Jigjid et al., 2009). Vachirarojpisan et al. (2004) also identified a relationship between S-ECC in children aged 15–19 months, and high numbers of mutans streptococci. The current investigation differs from many previous investigations in that the viable counts of supragingival plaque bacteria in S-ECC were obtained from the dental plaque overlying the carious teeth. This is arguably the most relevant location in terms of functional importance. Additionally, salivary microbial counts were also performed, providing additional information about the distribution of the oral microbiota giving information on the usefulness of saliva as a paradigm of supragingival plaque for compositional analyses. As well as supragingival plaque, which represents bacteria on the surface of the enamel, degraded dentine was also sampled. This comprises material sampled from the inside of carious lesions and therefore includes bacteria that have invaded the tooth during the advancement of the cariogenic process. The bacterial composition of supragingival plaque overlying teeth affected by caries was microbiologically similar to degraded dentine (**Figure 1**). However, counts of the cavity-implicated lactobacilli obtained from dental plaque and saliva were significantly higher in the diseased group whilst total streptococci were elevated in dental plaque but not in saliva. This observation contrasts with a study conducted by Gizani et al. (2009) where salivary streptococcal species were detected on the tooth surface more often than in saliva samples using DNA hybridization techniques.

With respect to familial factors, in accordance agreement with previous studies (Vachirarojpisan et al., 2004; Tiano et al., 2009), mother’s education level was significantly lower in the S-ECC group than in the CF group (*p* < 0.05) (data not shown). Vachirarojpisan et al. (2004) also reported a significant relationship between S-ECC in children aged 15–19 months old, and lower family income.

Salivary bacterial communities were significantly differentiated in terms of familial relationships (**Figure 3**) which corresponds to a previous study that reported a similarity range of between 74 and 94% in the salivary oral bacteria of mother-child pairs (Li et al., 2007b). Additionally, in the current study, the salivary and plaque microbiotas of sibling-child pairs were also investigated to assess the possibility of consortial transmission between siblings. DNA profiles derived from S-ECC children and their siblings were significantly differentiated in terms of the familial relationship. Additionally, bacterial viable counts were similar among family members except for lactobacilli where CF subjects had significantly lower numbers than their family members, while S-ECC subjects had a greater number of lactobacilli than their family members but this difference did not reach statistical significance (**Figure 6**). Importantly, children with S-ECC had significantly higher lactobacilli counts than CF children (*p* < 0.05), while counts of lactobacilli in mothers of children with S-ECC were not significantly higher than those of CF children’s mothers. This contrasts with a previous study (Al Shukairy et al., 2006), who found a difference for *S. mutans* counts, however, this was not statistically significant. Conversely, a Saudi Arabian study reported a significant relationship between mother-child pairs in the S-ECC group with respect to the numbers of salivary *S. mutans* (Al Shukairy et al., 2006). There was a significant relationship (*p* < 0.05) between numbers of salivary facultative anaerobes and streptococci between parents and child; and numbers of salivary anaerobes between sibling and child in this study. These results are in agreement with several previous reports (Emanuelsson et al., 1998; Redmo Emanuelsson and Wang, 1998; Li et al., 2007b) regarding the similarity and transmission of oral bacteria from mother or primary care giver to child and the cross relation between siblings (Tuite-McDonnell et al., 1997; Tinoco et al., 1998; Kohler et al., 2003). These results support the hypothesis that oral bacteria may be transmitted among family members. A more recent study of S-ECC in mother-child pairs reported a 41% maternal transmission of mutans streptococci (Mitchell et al., 2009) with over 70% of PFGE genotypes being shared between mother and child. The significance values generated through MDS analyses in the present study suggest transmission of oral bacteria between family members that is in accordance with plaque index scores and bacterial count data.

## Conclusion

The taxonomic composition of the oral microbiota within saliva and supragingival plaque could not be readily differentiated using MDS analysis of PCR-DGGE profiles between CF individuals and S-ECC patients. However, according to culture data, lactobacillus levels were considerably higher in children with S-ECC. The consumption of additional sweetened drinks was not correlated with S-ECC, but overnight use of a feed bottle was significantly associated with this condition A future study including a larger number of volunteers is required to confirm these observations. Oral bacterial DNA profiles could be differentiated based on familial relationships. Thus, as well as diet, the etiological contribution and potential for intervention associated with highly cariogenic microorganisms transferred from mother to child is worthy of further exploration.

## Author Contributions

KK collected the samples, performed the laboratory based analyses, and co-wrote the manuscript. RL co-wrote and contributed to data analysis. RT designed the study, supervised the sample collection, and studied the approval in Thailand. AM designed the study, supervised the project, performed the data analysis, and co-wrote the manuscript.

## Conflict of Interest Statement

The authors declare that the research was conducted in the absence of any commercial or financial relationships that could be construed as a potential conflict of interest.
